# The Influence of Entrepreneurial Education and Psychological Capital on Entrepreneurial Behavior Among College Students

**DOI:** 10.3389/fpsyg.2021.755479

**Published:** 2021-11-17

**Authors:** Jun Cui

**Affiliations:** School of Education Science, Nanjing Normal University, Nanjing, China

**Keywords:** entrepreneurial education, psychological capital, entrepreneurial behavior, college students, impact

## Abstract

This research investigated the influence of entrepreneurial education (EE) on entrepreneurial behavior (EB) through psychological capital (PC). A cross-section survey data of 1,405 college students in China were used to test the proposed hypothesis based on human capital theory and PC literature. The research found that EE had direct effects on EB and on all four sub-constructs (hope, self-efficacy, resilience, and optimism) of PC, and that only self-efficacy positively correlated with EB and mediated the relationship between EE and EB while the other three components of PC did not. These findings contribute to the understanding of both educational and psychological effects on EB. The study also has practical implications for policymakers, managers, and educators in entrepreneurial education.

## Introduction

Various countries are vigorously promoting entrepreneurship strategies because entrepreneurship is a critical way to accelerate innovation, increase economic growth, reduce the unemployment rate, and keep social stability (Ho et al., 2018). Entrepreneurship is perceived as a process that begins with entrepreneurial intention and finishes with taking action to engage in entrepreneurial activities (Gieure et al., 2020). Therefore, the actual entrepreneurial activity, or entrepreneurial behavior (EB), has been gained extensive attention from the academic community, policymakers, and practical educators (Nowiński et al., 2020).

Understanding the drivers of EB is particularly important as it can help to improve the effectiveness of entrepreneurial and educational initiatives. In literature, both external factors and internal factors influence entrepreneurial action and behavior (Franke and Lüthje, 2004). The former includes macro-environmental factors, such as the policy and ecosystem of economic development in a nation, and meso-environmental factors, such as education, experience, and family background. The latter always refers to micro-individual factors including entrepreneurial intention (EI).

In terms of internal factors, EI is a widely used variable to predict entrepreneurial behavior based on planned behavior theory (Ajzen, 1991). Although studies support the intention-behavior relationships, the predictive power of EI for EB is relatively weak in the entrepreneurship context (Martin et al., 2013; Rauch and Hulsink, 2015). Existing literature found that the explained variance of EI for EB is only around 18–27% (Fayolle and Liñán, 2014). This suggests that EI does not always translate into EB, and we should not rely solely on EI (Kautonen et al., 2013; Shirokova et al., 2016). Thus, from the perspective of internal predictors, it is necessary to consider other individual factors beyond EI, such as psychological factors. However, few studies have examined the evidence from psychological factors in entrepreneurship education situations (Yueh et al., 2020). For example, Liu et al. (2019) investigated the relationship between role model stories and entrepreneurial intention mediated by entrepreneurial passion as a psychological factor. Another example is Wei’s et al. (2019) work which identified self-efficacy and emotion regulation (an affective dimension) to influence entrepreneurial learning from failure.

Indeed, the psychological factor is beneficial to the development of entrepreneurial competencies and can stimulate students to be involved in entrepreneurial actions (Yueh et al., 2020). Psychological capital (PC) is a central construct in psychological factors, which has been proved an important antecedent of work engagement, job performance, organizational, and innovative behavior (Alessandri et al., 2018). In entrepreneurship contexts, PC is an eye-catching and promising variable to understand the complex entrepreneurship process (Tsai et al., 2020) because it has bearing on fostering creativity and maintaining entrepreneurship sustainability (Tang, 2020). Although researchers investigated the association of PC with EI (Mahfud et al., 2020; Maslakcı et al., 2021), there is insufficient evidence on the linkage of PC with entrepreneurial behavior. Thus, the extant literature lays an obvious research gap in individual drivers of entrepreneurial behavior.

Furthermore, concerning external factors, entrepreneurial education (EE) has been identified as a trigger of entrepreneurial intention and mindset (Bae et al., 2014; Cui, 2021; Cui et al., 2021). Empirical studies confirm that EE can exert a positive impact on entrepreneurial behavior (Ployhart and Moliterno, 2011; Rauch and hulsink, 2015). Nevertheless, there is still little evidence on how EE, directly or indirectly, impacts entrepreneurial behavior in various contexts, particularly in Chinese higher education settings. For example, whether the educational and psychological factors coherently drive the entrepreneurial behavior? There is scarce literature concerned with the mediating role of psychological capital of college students in the relationship between EE and EB. Thereafter, we need more research to verify such an influencing mechanism.

To fill the above gaps, the present study invokes human capital theory (HCT) and psychological capital literature to develop a mediating model. The purpose of this research was to investigate whether not only the external factor (entrepreneurial education) but also a novel internal factor (psychological capital) is associated with entrepreneurial behavior and to explore the mediating role of psychological capital in the EE-EB link under the context of Chinese higher entrepreneurship education. By addressing this, we believe that this study makes important contributions to the understanding of the psychological mechanism on entrepreneurial behavior of college students, and further how entrepreneurial education and psychological capital jointly influence entrepreneurial behavior.

## Theoretical Framework

**Figure 1:**
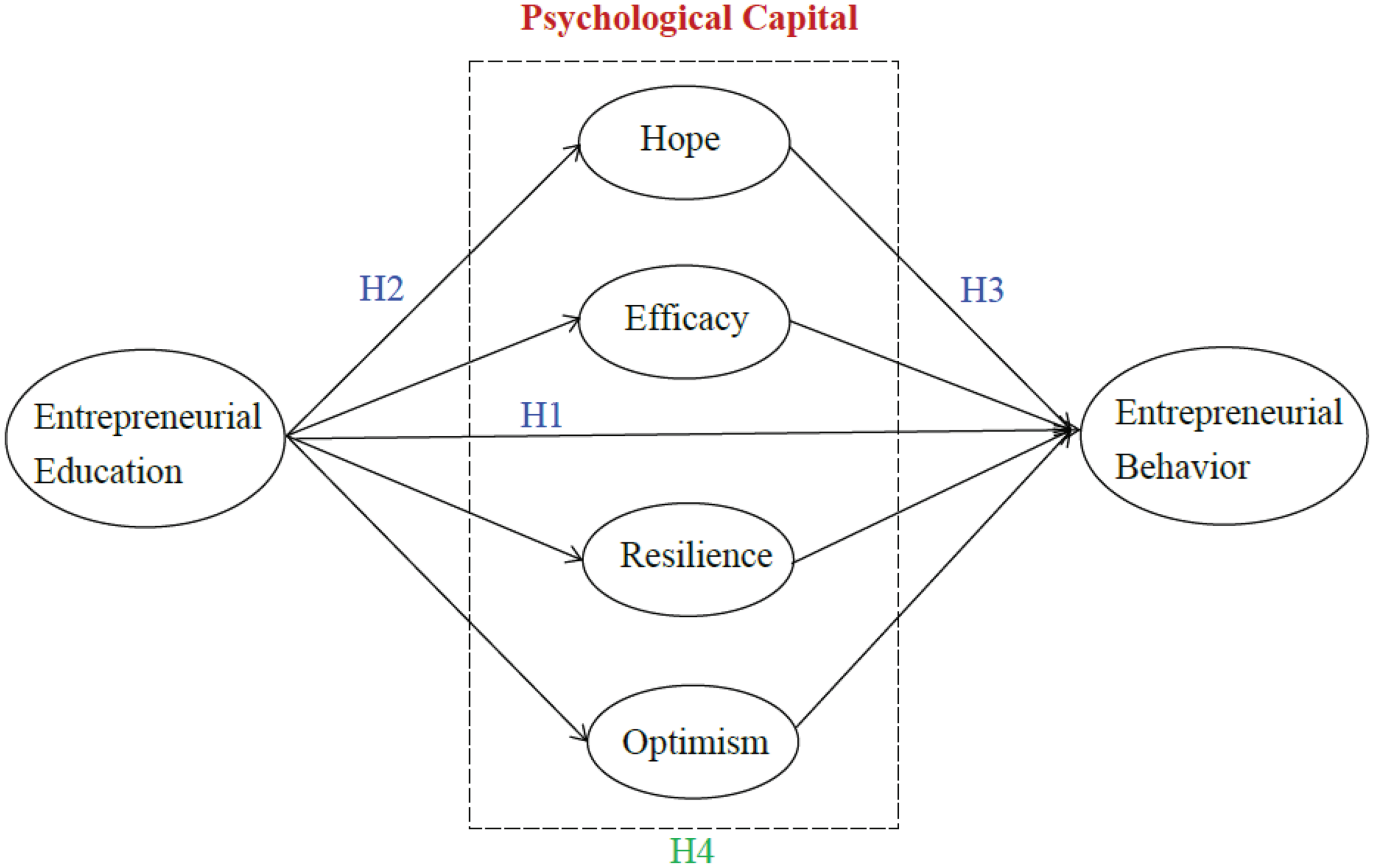
The hypothesized model. H1, H2, and H3 are hypotheses on direct effects; H4 is on mediating effects.

Building on HCT and psychological capital literature, we developed a research model to explain the influencing mechanism on entrepreneurial behavior in a higher education context. As illustrated in Figure 1, the factors that we theorize influence entrepreneurial behavior are not only the educational factor but also the psychological factors. Underpinning by HCT, we hypothesize that students’ entrepreneurial behavior is predicted by their involvement in entrepreneurial education (H1). To understand the role of psychological capital, we also investigate the relationships between entrepreneurial education, psychological capital, and entrepreneurial behavior (H2 and H3), as well as the mediating role of psychological capital (H4).

### Human Capital Theory

To explain the relationship between entrepreneurial education and entrepreneurial behavior, we ground on HCT, which was initially proposed to study the value of education on economic value through individuals’ development of knowledge and skills (Becker, 1964). This theory further differentiates human capital investments from human capital assets (Martin et al., 2013) with a dynamic view, and the former refers to the input, such as education, training, and experience, while the latter refers to the output including knowledge, skills, and abilities. Human capital is relevant to entrepreneurship because it can help individuals to discover and utilize the entrepreneurial opportunity by acquiring verified resources and to accumulate new knowledge and skills in launching and developing a new venture (Marvel et al., 2016).

In the context of entrepreneurial education, entrepreneurial courses and extra-curriculum activities are specific types of education and learning experiences that belong to human capital investment, and entrepreneurial behavior underpinning by specific competencies is a vital outcome of such education belongs to human capital assets (Martin et al., 2013). In the extant literature, human capital is the most-used theory for explaining the formation of entrepreneurial behavior in the higher education system (Dimov, 2017). Therefore, human capital is an ideal theory to explain the relationship between entrepreneurial education perceived by students and their subsequent behavior on campus.

### Psychological Capital in the Entrepreneurship Context

Psychological capital (PC) has been recognized as a core construct in the field of positive organizational behavior within the positive psychology discipline. PC was defined by Luthans and colleagues to describe “an individual’s positive psychological state of development characterized by the need for achievement of goals to succeed; having the confidence to act upon challenging tasks; sustaining and bouncing back when beset by problems; and making a positive attribution.” (Luthans et al., 2007, p.542).

In nature, PC is a positive state-like resource rather than trait-like. This means that PC is malleable, open to development, and easy to be changed, while a trait-like resource is more fixed, stable, and difficult to change (Luthans et al., 2017). Thus, PC can be expected and growing over time through targeted training and short interventions. Existing literature suggests that PC is theoretically influenced by conation, positive cognitive appraisals, emotions, and social mechanisms (Youssef and Luthans, 2013; Youssef-Morgan and Luthans, 2013).

Based on its definitions, PC is a second-order construct consisting of hope, self-efficacy, resilience, and optimism. Although the four first-order components have their own characteristics, they share a common commonality that is “positive appraisal of circumstances and probability for success based on motivated effort and perseverance” (Luthans et al., 2007, p.550). Such positive appraisal is measured to capture an individual’s sense of control, intentionality in the process of goal pursuit. To date, the core construct of PC combined four dimensions has been empirically recognized with valid measures.

Specifically, *hope* is defined as a state of motivation in achievements based on goal orientation and action pathways to success (Snyder et al., 1991); *efficacy* is one’s confidence in abilities to successfully perform a specific task within resources (Stajkovic and Luthans, 1998); and *resilience* is depicted as a positive adaption to rebound back from obstacles, adversity, and failures in a risky situation (Luthans, 2002; Masten et al., 2009). *Optimism* reflects positive attributes and expectations for the future even in face of negative events or frustration (Seligman, 1998). Synergistically, the four resources of PC can help a person to hold reasonable appraisals and maintain a positive state of psychology leading to successful achievement under complicated conditions.

PC is relevant in the entrepreneurship context. It contributes to sustainable entrepreneurship (Kyrö, 2015). Entrepreneurial individuals intend to generate new ideas and make them happen. This necessitates them to undertake actions through new opportunities. To obtain the goal, entrepreneurs need to utilize natural and social resources by the proper application of various capital, including human capital, such as the PC. Research has found that PC can facilitate an individual’s creativity generation, communication with stakeholders, adaption to dynamic changes in complex situations, which lead to personal development and sustainable success in entrepreneurship (Tang, 2020). This is because the state-like PC determines the emotional intelligence of an individual and motivates individuals to achieve a particular goal with entrepreneurial skills in a decision-making process. Thus, PC is strongly relevant to an entrepreneurial context (Tsai et al., 2020).

## Hypothesis Development

### Entrepreneurial Education and Entrepreneurial Behavior

Entrepreneurial behavior (EB) is expressed by individuals combing self-determination, self-efficacy, and self-identity based on specific values, beliefs, and needs (Kirkley, 2016). Extant literature notes that there may be important relationships between EE and entrepreneur outcomes. For example, Unger et al. (2011) found a stronger relationship between task-related human capital, than general human capital, and entrepreneurial performance. Considering EB is a fewer distal outcome than new venture success, we expect EE has an impact on EB in a higher education context.

Human capital theory is an appropriate theory to explain the impact of EE on EB. Researchers note that there may be important positive links between EB and human capital assets including entrepreneurial behavior (Martin et al., 2013). Based on HCT, the EB is the outcome of the EE just as the human capital investment becomes the assets in human capital (Martin et al., 2013). Also, in Marvel’s et al. (2016) review literature, education is one of the most-used common human capital constructs, and entrepreneurial behavior is a task-related outcome and dependent construct in the entrepreneurial process. Moreover, empirical research supports the links between EE and EB in a higher education context. For example, Rauch and Hulsink’s (2015) study confirmed that participation in EE has a positive effect on EB suggesting EE emphasizes increasing EB. Therefore, we propose:

*Hypothesis 1 (H1):* Entrepreneurial education (EE) is positively related to entrepreneurial behavior (EB).

### Entrepreneurial Education and Psychology Capital

Psychological capital (PC) is a changeable mental state and can be shaped in personal stages of growth and development. Instead of trait-like, PC is state-like feelings that are malleable (Luthans et al., 2007). Further, the plasticity and openness to change is the distinguishing characteristic of PC (Luthans and Youssef-Morgan, 2017). As such, PC can be developed within positive thinking patterns that can replace deep-rooted beliefs over time. In addition, according to HCT, as an emotional capital of a human, PC can be cultivated by education and training. To date, the PC training model has been developed online and offline (Luthans et al., 2008; Luthans and Youssef-Morgan, 2017). This implies that the nature of PC lies at its malleability which is easy to change and be shaped through educational intervention. Therefore, in the entrepreneurship context, entrepreneurial education should have an impact on PC in theory.

There is empirical evidence that PC develops over time (Avey et al., 2010) and that PC can be developed through education and training interventions (Dello Russo and Stoykova, 2015). For example, Luthans et al. (2010) tested whether PC can be shaped using an experimental design and provided beginning empirical evidence on the development of PC via short training interventions. Although there is a scarcity of research on the impact of EE on PC as an individual variable, there is evidence on the education impact on the components of PC, such as efficacy and optimism. For example, various studies supported EE is associated with self-efficacy and could enhance entrepreneurial self-efficacy (Bae et al., 2014). Also, Crane (2014) suggests a positive link between EE and dispositional optimism. Thus, we suggest:

*Hypothesis 2 (H2):* Entrepreneurial education (EE) is positively related to psychological capital: (a) hope, (b) self-efficacy, (c) resilience, and (d) optimism.

### Psychology Capital and Entrepreneurial Behavior

Research suggests that behavioral outcomes of PC are of critical importance (Newman et al., 2014). In a meta-analysis review, Avey et al. (2011) conclude that PC negatively relates to undesirable behavior and positively to desirable behaviors of employees. In the entrepreneurship context, the study hypothesized and confirmed that PC has a positive effect on start-up intention among young start-up entrepreneurs (Jin, 2017). According to the theory of planned behavior, intention can predict behavior, so PC may theoretically relate to entrepreneurial behavior. Also, PC is found to have a positive impact on EB in terms of environmental perception and opportunity recognition in a new generation of migrant workers in China (Ma et al., 2018).

In the Chinese context, empirical evidence shows that entrepreneurial PC significantly correlated with deviant innovation behavior (Xu and Zhao, 2020) and with entrepreneurial behavior in terms of entrepreneurial opportunity ability of employees (Gao et al., 2020). Also, as a representative capital of entrepreneurs, PC is reported to have an effect on new venture performance (Wang et al., 2019), creative innovation behavior and enterprise performance (Gao et al., 2020), belonging to the domain of entrepreneurial behavior. Synthesizing the above evidence, we propose:

*Hypothesis 3 (H3):* Psychological capital (a) hope, b) self-efficacy, c) resilience, and d) optimism) is positively related to entrepreneurial behavior (EB).

### The Mediating Role of Psychology Capital

Extant research shows that the components of PC could be learned and strengthened through relevant interventions (e.g., Bakker et al., 2017) and that PC affects people’s behavior in many ways (Donaldson, 2013). Although there is less direct evidence on the mediating role of PC in the link of entrepreneurial education and entrepreneurial behavior, studies reported that both entrepreneurial capitals and PC are significant predictors of entrepreneurial success (Martin et al., 2016; Zhao et al., 2020), that PC is related to entrepreneurial intention as a whole (Contreras et al., 2017), and that entrepreneurs’ PC could explain significant variance in new venture performance (Hmieleski and Carr, 2008). This indicates that PC can be derived from entrepreneurial education and should in turn affect entrepreneurial behavior.

In theory, PC is a state of feelings, moods, or emotions, and thus, the mediating role of PC between education and behavior can be explained from the emotional perspective. Learning theory holds that learning is a complex process acquired through the integration of thoughts, emotions, and actions (Jarvis, 2006), and entrepreneurship theory posits that entrepreneurship is an emotional journey in nature. In higher entrepreneurship education, emotion-based development is an important impact outcome of entrepreneurial learning (Gibb, 2002; Nabi et al., 2017). Research findings indicate that different educational designs of creating value trigger emotional events of frequent interaction with the outside world including the affection of happiness, frustration, anger, and despair (Lackéus, 2014). Similarly, entrepreneurship education enhances entrepreneurial intention mediated by students’ emotions including optimism that is an element of PC. In this vein, we suggest that:

*Hypothesis 4 (H4):* Psychological capital (a) hope, b) self-efficacy, c) resilience, and d) optimism) plays a mediating role in the relationship between EE and EB.

## Materials and Methods

### Data Collection and Sample

In this study, data were collected from 15 higher education institutions in Jiangsu province, China, using convenience sampling methods. In entrepreneurship education studies, convenience sampling is prevalent (e.g., Nowiński et al., 2019). In this vein, Coviello and Jones (2004) argue that although non-probability sampling has generalizability limitations, the method still results in quality data when the response rates and participation levels of samples are high. Jiangsu was chosen as the province has implemented an enterprise and EE strategy in colleges and universities to promote regional innovation and entrepreneurship. Altogether 15 institutions were selected because of their implementation of entrepreneurship education. Therefore, the participants are appropriate to provide rich information in the context of entrepreneurship education.

The voluntary and anonymity were guaranteed during the data collection with a response rate of 81.09%. Finally, a total of 1,405 valid samples were used in this study. Among the samples, 50.6% were women, with 97.8% of students aged between 18 and 23; the proportion of students from year-one was 40.2%, year-two 36.8%, year-three 18.6%, and year-four 4.3%; the distribution of the field of study was as follows: 56.4% science and engineering and 43.6% humanity and social sciences.

### Measures

The measure in this study was adapted from existing literature. The scales were double-back translated from English to Chinese by two bio-linguistic academics to minimize method biases. The scales were pilot tested the scale on students from different institutions and revised according to their feedback.

#### Psychological Capital

*Psychological Capital* (PC) was measured adopt Luthans’ et al. (2007) influential measures in four sub-dimensions: hope, self-efficacy, resilience, and optimism, to capture the psychological and emotional state of college students. It was adapted to suit Chinese entrepreneurship education culture based on extant literature (Sinclair and Wallston, 2004; Zeffane, 2013; Crane, 2014; Sieger’s et al., 2014). In total, 17 items were used to measure the four components of the PC. Hope was assessed with four items, and a sample item was “Whether alone or working with others, I usually try my best.” Self-efficacy was measured in entrepreneurial situations with seven items, and a sample item was “I can become a leader and coordinator now.” Resilience was assessed with four items, for example, “I am willing to innovate to change the difficult environment.” Optimism was measured with three items, for example, “In uncertain times, I would expect the best.” Participants were asked to express agreement with each statement on 7-point Likert scale. Each sub-construct was scored by the average value ranging from 1 to 7.

#### Entrepreneurial Behavior

*Entrepreneurial behavior* (EB) was measured based on Rauch and Hulsink’s (2015) work by three questions to capture the depth and breadth of college students’ EB. The first question was a “yes” or “no” binary question and asked whether the participant had established their own business. If “yes,” then scored 5. If “no,” then jumped to the second question that was also a binary question asking whether the students are currently trying to start a new business. If “yes,” then scored 4; If “no,” then jumped to the third question of a multiple-choice question to ask respondents to select any behavioral activities in entrepreneurship from a list of 18 items for behavioral activities and 1 item for “nothing at all” (scoring 0). Among them, 18 behaviors were related to venture creation (e.g., saving money to invest in a business, started marketing or promotional activities), scoring 1 for selecting 1–6 behaviors, 2 for 7–12 behaviors, and 3 for above 12 behaviors.

#### Entrepreneurial Education

*Entrepreneurial education* (EE) was measured adopted from existing literature with altogether 12 “yes” or “no” binary items (Sieger’s et al., 2014; Arranz’s et al., 2017). Among them, two items focused on attendance in the compulsory course and optional course already used in the survey by Sieger’s et al. (2014). Other 10 items were created by Arranz’s et al. (2017) centered on EE extracurricular activities, such as entrepreneurship clubs, face-to-face communication with an entrepreneur, and entrepreneurial incubation project. Students were asked to answer whether they have participated in these courses and educational activities. We scored 0 or 1 for each item. Consequently, the score of variable EE was summed up by each score of the item, capturing the degree of students’ involvement in EE.

#### Control Variables

Students’ g*ender*, *year of study*, *the field of study,* and *institution type* were controlled in dichotomous variables (Nabi’s et al., 2017). Moreover, previous studies have indicated that the initial state of entrepreneurial intention may affect the current state of intention (Fayolle and Gailly, 2015). The initial level of EI was also used to control personal factors in Cui’s et al. (2021) study. Thus, in this study, *the initial level of entrepreneurial intention* was controlled. Students were asked to assess their initial degree of EI on a single 7-point scale from 1 (very low level) to 7 (very high level).

## Analyses and Results

### Measurement Reliability and Validity

In this study, the variable of psychological capital is a composite construct with reflective measures. The reliability is typically assessed through Cronbach’s alpha (α) and composite reliability (CR). In Table 1, all values of α and CR exceed the 0.7 thresholds (MacKenzie et al., 2011) ranging from 0.822 to 0.952. Further, using confirmatory factor analysis (CFA), all the loadings are bigger than 0.7 ranging from 0.775 to 0.899. These show that the construct was highly reliable.

**Table 1:** Reliability, validity, descriptive statistics, and correlations.

Variable	*α*	CR	AVE	Mean	SD	1	2	3	4	5	6	7	8	9	10
1. Hope	0.923	0.923	0.800	4.857	1.388	(0.894)									
2. Efficacy	0.952	0.952	0.740	4.302	1.300	0.516^**^	(0.860)								
3. Resilience	0.920	0.920	0.742	4.715	1.285	0.848^**^	0.612^**^	(0.861)							
4. Optimism	0.822	0.822	0.607	4.679	1.250	0.731^**^	0.562^**^	0.738^**^	(0.779)						
5. EB				2.550	2.146	0.036	0.164^**^	0.086^**^	0.059^*^						
6. EE				1.390	1.772	0.030	0.176^**^	0.058^*^	0.065^*^	0.069^**^					
7. Gender				0.510	0.500	0.038	−0.145^**^	−0.001	0.011	−0.063^*^	−0.135^**^				
8. Field				0.440	0.496	−0.018	−0.018	−0.031	−0.001	−0.013	−0.004	0.265^**^			
9. Year				1.870	0.864	0.033	0.007	0.046	0.054^*^	0.159^**^	0.006	0.154^**^	0.060^*^		
10. Institution				0.350	0.477	−0.055^*^	0.115^**^	−0.029	−0.031	0.203^**^	0.115^**^	−0.239^**^	−0.125^**^	−0.339^**^	
11. IEI				3.770	1.845	0.216^**^	0.407^**^	0.266^**^	0.227^**^	0.168^**^	0.256^**^	−0.246^**^	−0.112^**^	−0.149^**^	0.237^**^

α, Cronbach’s alpha; CR, composite reliability; AVE, average variance extracted; SD, standard deviation; EB, entrepreneurial behavior; EE, entrepreneurial education; IEI, the initial level of entrepreneurial intention; The diagonal values (in brackets) are the square root of corresponding AVE, and the values on triangle elements are correlations among the variables. n=1,405. ^*^p<0.05; ^**^p<0.01.

The validity was examined by the average variance extracted (AVE). Table 1 presents that all AVE scores (from 0.607 to 0.800) exceed the 0.5 cutoff value (Schmiedel et al., 2014), suggesting a good convergent validity. The square roots of AVE in the diagonal elements are larger than the correlations with the remaining constructs in the off-diagonal elements, indicating the discriminant validity is established. Further, the CFA showed that the four-factor model was better than other constraining models with satisfactory model fit indices [CFI=0.981, TLI=0.977, RMSEA=0.051(0.047, 0.056), SRMR=0.029] which indicates sufficient discriminant validity.

Moreover, a common latent factor technique was used to test for common method bias (Podsakoff’s et al., 2003). In Table 2, the results find that the fitting index of the model after control did not change substantially (Δ*χ*^**2**^=47.326, Δdf=11, *p*>0.05; ΔCFI=0.002, ΔTLI=0.000, ΔRMSEA=0.001, ΔSRMR=0.006), suggesting that common method bias did not affect the results of this study. Finally, collinearity problems were checked by computing the variance inflation factor (VIF). The results revealed that all the VIFs ranging from 1.089 to 4.449 are below the threshold value of 5 (Hair et al., 2011), suggesting that there was no collinearity issue.

**Table 2:** Model fit indices for measurement model.

Model	*χ* ^2^	df	*χ*^2^/df	∆*χ*^2^/df	CFI	TLI	RMSEA	SRMR
HP+EC+RL+OT	5976.801	117	51.083	—	0.736	0.693	0.189[0.185,0.193]	0.122
HP+RL+OT, EC	1145.448	116	9.875	41.208	0.954	0.946	0.079[0.075,0.084]	0.045
HP, RL+OT, EC	820.261	114	7.195	2.680	0.968	0.962	0.066[0.062,0.071]	0.036
HP, RL, OT, EC	520.998	111	4.693	2.502	0.981	0.977	0.051[0.047,0.056]	0.029
HP, RL, OT, EC, CMV	473.672	100	4.737	0.044	0.983	0.977	0.052[0.047,0.056]	0.035

*The four factors in the measurement model are: HP hope, EC efficacy, RL resilience, OT optimism. CMV common method variance*.

### Hypothesis Testing

**Figure 2:**
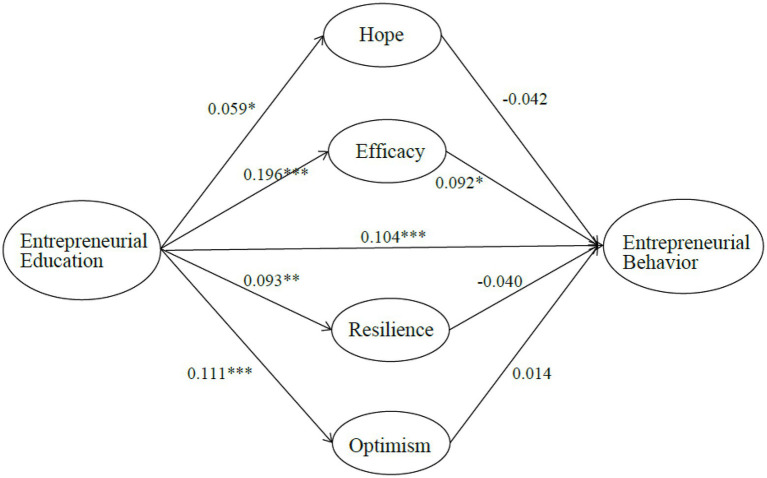
Coefficients and significance of the structure model. Control variables are gender, the field of study, year of study, institution type, and the initial level of entrepreneurial intention. *n*=1,405; ^*^p<0.05, ^**^p<0.01, ^***^p<0.001.

MPLUS software was used to test the proposed hypotheses in a four-mediator model with control variables. The path coefficients (*β)* and the *p* values are summarized in Figure 2.

First, the direct effect of entrepreneurship education (EE) on entrepreneurial behavior (EB) was tested in terms of Hypothesis 1, and results are presented in Table 3. Among the control variables, it is noted that gender negatively influences EB, while initial entrepreneurial intention (IEI) positively influences EB. The results show that EE is positively related to EB (*β*=0.104; *p*<0.001), and thus, H1 is supported.

**Table 3:** Direct effects from entrepreneurial education to entrepreneurial behavior.

DV	HP		EC		RL		OT		EB	
	Estimate	S. E.	Estimate	S. E.	Estimate	S. E.	Estimate	S. E.	Estimate	S. E.
Gender	0.087^**^	0.030	−0.058^*^	0.027	0.060^*^	0.030	0.057	0.031	−0.071^*^	0.028
Field	−0.029	0.028	0.028	0.025	−0.032	0.028	−0.007	0.030	0.035	0.028
Year	0.018	0.030	0.045	0.028	0.039	0.028	0.048	0.031	0.046	0.026
Institution	−0.105^**^	0.033	0.003	0.027	−0.097^**^	0.032	−0.086^**^	0.032	0.039	0.029
IEI	0.257^***^	0.031	0.366^***^	0.028	0.291^***^	0.031	0.267^***^	0.032	0.192^***^	0.030
EE	0.059^*^	0.028	0.196^***^	0.026	0.093^**^	0.028	0.111^***^	0.030	0.104^***^	0.029
HP									−0.042	0.051
EC									0.092^*^	0.038
RL									−0.040	0.054
OT									0.014	0.047
RV	0.926^***^	0.015	1.155^***^	0.066	1.398^***^	0.072	1.291^***^	0.085	0.899^***^	0.018
R^2^	0.640		0.205		0.090		0.070		0.090	

The value of estimate is the standardized results, and RV means residual variances. EB entrepreneurial behavior, EE entrepreneurship education, IEI the initial level of entrepreneurial intention, HP hope, EC Efficacy, RL Resilience, OT Optimism. ^*^p<0.05, ^**^p<0.01, ^***^p<0.001.

The direct effects of EE on the four facets of psychological capital (PC) were tested, corresponding to Hypotheses 2a, 2b, 2c, and 2d. Results are also shown in Table 3. Among the control variables, it is noted that gender, institution, and IEI influence four PCs (positively or negatively). The results revealed that EE positively influences all four PCs, namely hope (*β*=0.059; *p*<0.05), self-efficacy (*β*=0.196; *p*<0.001), resilience (*β*=0.093; *p*<0.01), and optimism (*β*=0.111; *p*<0.001). These findings are consistent with H2.

Also, the direct effects of four PCs on EB, corresponding to Hypotheses 3a, 3b, 3c, and 3d, were tested. In Table 3, the results indicate that EB is influenced only by self-efficacy (*β*=0.092; *p*<0.05) but the coefficients of hope (*β*=−0.042; *p*>0.05), resilience (*β*=−0.040; *p*>0.05), and optimism (*β*=0.014; *p*>0.05) are not significant. Thus, H3b is supported but H3a, H3c, and H3d are not supported.

Then, the indirect effects of four mediators were tested. The results of the mediation analysis are shown in Table 4. The indirect effects of hope (*β*=−0.002), resilience (*β*=−0.004), and optimism (*β*=0.002) on the relationship between EE and EB are not significant (*p*>0.05). However, the specific indirect effect of self-efficacy (*β*=0.018; *p*<0.05) and the bootstrapping confidences [0.006, 0.031] is significant. This indicates that self-efficacy plays a mediating role in the EE-EB link, while the other three PCs do not. Therefore, H4b is supported and H4a, H4c, and H4d are not supported.

**Table 4:** Mediation test for the psychological capital.

DV	EB			
Specific indirect	Standardized estimate	S. E.	Two-tailed value of p	95% confidence interval
EE→HP→EB	−0.002	0.004	0.484	[−0.011, 0.001]
EE→EC→EB	0.018^*^	0.008	0.021	[0.006, 0.031]
EE→RL→EB	−0.004	0.005	0.487	[−0.014, 0.004]
EE→OT→EB	0.002	0.005	0.772	[−0.007, 0.011]
Total (c)	0.117^***^	0.029	0.000	[0.065, 0.161]
Direct (cʹ)	0.104^***^	0.029	0.000	[0.050, 0.147]
Total indirect (ab)	0.013^*^	0.007	0.049	[0.003, 0.026]

EB, entrepreneurial behavior; EE, entrepreneurship education; IEI, the initial level of entrepreneurial intention; HP hope, EC, Efficacy; RL, Resilience; OT, Optimism. ^*^p<0.05, ^**^p<0.01, ^***^p<0.001.

Next, the size of the indirect effects was examined. In Table 4, the total effect (c) of EE on EB is 0.117 (*p*<0.001), the direct effect (cʹ) is 0.104 (*p*<0.001), and the total indirect effect (ab) is 0.013 (*p*<0.05), suggesting that mediators altogether contribute 11.11% (ab/c) for the total effect in the EE-EB link.

Finally, the research model piecewise was tested to confirm the robustness. Only one mediator at a time was added in the main effect of EE on EB. Mediation analyses were conducted four times. These four separate results are consistent with the results from the overall model for our hypothesis tests, indicating that our findings are robust.

## Discussion

### Key Findings

The research finds that entrepreneurial education has a positive influence on the entrepreneurial behavior of students in higher education. This is in line with Rauch and Hulsink’s (2015) finding that EE programs positively affect university students. HCT can explain the relationship between EE and EB, in which the former is a capital investment and the latter belongs to capital assets (Ployhart and Moliterno, 2011). Based on HCT, entrepreneurship education provides an individual with knowledge, skills, and experiences enhancing individuals’ competence to identify and exploit entrepreneurial opportunities that will lead to a greater likelihood of entrepreneurial behavior (Souitaris et al., 2007).

This study finds that entrepreneurial education impacts four sub-dimensions of psychological capital. This result provides empirical support with the view that PC is open to development and could be learned through educational interventions (Luthans and Youssef-Morgan, 2017; Bakker et al., 2017). Moreover, according to the path coefficients, among the components of PC, the result finds that the relationship between EE and self-efficacy is the strongest, the relationship between EE and optimism is the second, and the link with hope and resilience is relatively weaker although the coefficients are significant. Existing literature has confirmed that EE could affect and increase students’ entrepreneurial self-efficacy (Bae et al., 2014). This finding implies that the outcome of self-efficacy and optimism is more impactful than hope and resilience by entrepreneurial education.

The research also finds that only self-efficacy affects entrepreneurial behavior, while hope, resilience, and optimism do not have such an effect. This indicates the influence of PC’s components on the outcome is unbalanced. This could be explained by three possible reasons. Basically, this could be interpreted by the weighted role of self-efficacy itself. For example, a meta-analysis of self-efficacy confirmed the correlation between self-efficacy and work-related performance (Stajkovic and Luthans, 1998). Secondly, although studies have reported that PC is a predictor of start-up intention, innovative behavior, opportunity recognition, and new venture performance (e.g., Alessandri et al., 2018; Ma et al., 2018; Wang et al., 2019), these outcome variables are not entrepreneurial behavior itself which means part of behaviors and actions in entrepreneurial activities in this study. Thirdly, this research is emersed in the context of entrepreneurship education in Chinese higher education which is different from some studies in the literature, thus leading to a novel finding.

Accordingly, this study finds that only self-efficacy plays a mediating role in the relationship between EE and EB but the mediating role of the other three elements of PC are all not significant. This finding highlights the role of self-efficacy in the EE-EB link, suggesting that self-efficacy is relatively important in the process from education to behavior in the entrepreneurship situation, compared with the other three elements of PC. In theory, hope, resilience, and optimism are states of emotional attitudes and feelings that may help to the formation of intention but are not necessarily yield the actual behaviors related to entrepreneurship. However, self-efficacy is the individual’s confidence in owning abilities to successfully execute intentions (Luthans et al., 2017) which can promote the individual’s entrepreneurial passion and is more likely to result in real actions in the process of entrepreneurship (Baum and Locke, 2004).

It is worth noting that the role of the IEI should be stressed. The results show that although entrepreneurial education and self-efficacy relate to entrepreneurial behavior, the relationship between self-efficacy and behavior is weak (*β*=0.092). However, the effect of the *initial level of entrepreneurial intention* on entrepreneurial behavior is stronger (*β*=0.192). This finding may shed light on the contribution of IEI on the stimulation of entrepreneurial behavior among college students which is in line with Fayolle and Gailly’s (2015) work.

### Practical Implications

The research findings have practical implications for the effectiveness assessment of entrepreneurial education. On one hand, the result suggests a direct and positive impact of entrepreneurial education on the entrepreneurial behavior of students on a university campus. As an intentional and self-determined behavior, entrepreneurial behavior is helpful to students’ employability and future entrepreneurship. In this sense, this study confirms the value of entrepreneurial education by the government and universities, and encourage policymakers to continuously support higher education institutions with funding for the research and initiatives of entrepreneurial education, especially the innovation of curriculum course and extracurricular activities.

On the other hand, this study revealed that psychological capital is a crucial benefit from entrepreneurial education. This implies that university entrepreneurial programs should include psychological capital in the assessment framework so that to evaluate the effectiveness of entrepreneurial education in a more theory-based approach. Accordingly, teachers and instructors should consider psychological capital development as one of the basic objectives of courses and co-curricular and stimulate students to strengthen their state of psychological capital during the teaching process. It is worth noting that, as our findings highlight the mediating role of self-efficacy in the impact mechanism from entrepreneurial education to behaviors of students, educators should particularly capture students’ changes in entrepreneurial self-efficacy and the following behavior among the psychological capital from entrepreneurial courses and activities.

## Conclusion

This study aimed at unpacking the educational and psychological mechanism on entrepreneurial behavior of university students. Using HCT and psychological capital framework, we conceptualized a mediating model connecting entrepreneurial education and entrepreneurial behavior. Based on survey data from 15 higher education institutions in China, we tested our hypothesis. The results confirmed the direct impact of entrepreneurial education on both entrepreneurial behavior and four elements (hope, self-efficacy, resilience, and optimism) of psychological capital. The results further established the mediating role of self-efficacy in the impact mechanism despite the non-indirect effects of hope, resilience, and optimism. These findings contribute theoretically to the existing literature.

### Theoretical Contributions

First, this study offers a model of the influencing mechanism on entrepreneurial behavior. We conceptualized and tested a mediating model integrating both entrepreneurial education and psychological capital based on students’ data under a context of higher entrepreneurship education in China. This model can explain not only the environmental factor (education) but also the psychological factors (capital) for the formation of entrepreneurial behavior in a specific situation. By this, the model contributes to our understanding of “how” entrepreneurial education affects entrepreneurial behavior of “whom” in “where.”

Second, this research reveals a divergent influence of psychological capital on entrepreneurial behavior. The findings confirm that, among four constructs of psychological capital, self-efficacy is the only significant and positive predictor for entrepreneurial behavior and that only self-efficacy has the mediating role in the development of entrepreneurial behavior. This provides a non-obvious nature of psychological capital to stimulate entrepreneurial behavior in a Chinese context and thus contributing to a nuanced understanding of the multi-facet role of different components of psychological capital in the prediction for entrepreneurial behavior.

Third, this study expands upon the impact type of entrepreneurial education by highlighting psychological capital. The study verifies that entrepreneurial education influences hope, self-efficacy, resilience, and optimism in a positive way. Understanding psychological capital is important because it represents the emotional aspects of the impact of entrepreneurial education, extending prior research dominated by cognitive aspects (e.g., entrepreneurial intention). Therefore, this research contributes to extending impact indicators of entrepreneurial education from a psychological angle.

Fourth, this research enriches the literature on psychological capital itself. The results suggest that entrepreneurial education can impact psychological capital, of which only the component of self-efficacy can in turn affect entrepreneurial behavior. This provides an explanation not only for the educational formation but also for the behavioral consequence of psychological capital. As such, it contributes to our understanding of how psychological capital works by unpacking its alternative antecedent and consequent.

### Limitations and Future Research

Firstly, this study used a self-report measurement for entrepreneurial behavior and entrepreneurial education of higher education students. Although the self-report approach is suitable to capture students’ education and behaviors, future research could use another objective method, such as observations on students’ engagement in entrepreneurial education and the real entrepreneurial actions at the campus.

Secondly, the research revealed unbalanced effects of four components of psychological capital which helps to our understanding of the multi-facet inner part of the entrepreneurial capital. However, this research design could not deeply explain the reason why hope, resilience, and optimism do not affect entrepreneurial behavior. Future research could explore some contextual factors (e.g., family background of the students) or other mediating variables (e.g., entrepreneurial intention and entrepreneurial passion), so that to explain the conditional effect of different psychological capital on entrepreneurial behavior.

Last, the sample of this study is only based on a province in China that generated qualified data. Future work may extend the sample to other areas in the country to make the sample more representative and could compare the differences from different areas on the impact of entrepreneurial education on entrepreneurial behavior via psychological capital among the students in higher education.

## Data Availability Statement

The datasets presented in this article are not readily available because the datasets are confidential as promised before the survey. Questions regarding the datasets should be directed to JC, jun.cui@njnu.edu.cn.

## Author Contributions

The author confirms being the sole contributor of this work and has approved it for publication.

## Funding

This research was funded by China National Social Science Foundation in Education under grant BIA170207.

## Conflict of Interest

The author declares that the research was conducted in the absence of any commercial or financial relationships that could be construed as a potential conflict of interest.

## Publisher’s Note

All claims expressed in this article are solely those of the authors and do not necessarily represent those of their affiliated organizations, or those of the publisher, the editors and the reviewers. Any product that may be evaluated in this article, or claim that may be made by its manufacturer, is not guaranteed or endorsed by the publisher.

## References

[ref1] Ajzen I. (1991). The theory of planned behavior. Organ. Behav. Hum. Decis. Process. 50, 179–211. doi: 10.1016/0749-5978(91)90020-T

[ref2] Alessandri G. Consiglio C. Luthans F. Borgogni L. (2018). Testing a dynamic model of the impact of psychological capital on work engagement and job performance. Career Dev. Int. 23, 33–47. doi: 10.1108/CDI-11-2016-0210

[ref3] Arranz N. Ubierna F. Arroyabe M. F. Perez C. Arroyabe F. D. (2017). The effect of curricular and extracurricular activities on university students’ entrepreneurial intention and competencies. Stud. High. Educ. 42, 1979–2008. doi: 10.1080/03075079.2015.1130030

[ref4] Avey J. B. Luthans F. Smith R. M. Palmer N. F. (2010). Impact of positive psychological capital on employee well-being over time. J. Occup. Health Psychol. 15, 17–28. doi: 10.1037/a0016998, PMID: 20063956

[ref5] Avey J. B. Reichard R. J. Luthans F. Mhatre K. H. (2011). Meta-analysis of the impact of positive psychological capital on employee attitudes, behaviors, and performance. Hum. Resour. Dev. Q. 22, 127–152. doi: 10.1002/hrdq.20070

[ref6] Bae T. J. Qian S. Miao C. Fiet J. O. (2014). The relationship between entrepreneurship education and entrepreneurial intentions: a meta-analytic review. Enterp. Theory Pract. 38, 217–254. doi: 10.1111/etap.12095

[ref7] Bakker D. J. Lyons S. T. Conlon P. D. (2017). An exploration of the relationship between psychological capital and depression among the first-year doctor of veterinary medicine students. J. Vet. Med. Educ. 44, 50–62. doi: 10.3138/jvme.0116-006r, PMID: 28206833

[ref8] Baum J. R. Locke E. A. (2004). The relationship of entrepreneurial traits, skill, and motivation to subsequent venture growth. J. Appl. Psychol. 89, 587–598. doi: 10.1037/0021-9010.89.4.587 15327346

[ref9] Becker G. (1964). Human Capital. New York, Columbia University Press.

[ref11] Contreras F. de Dreu I. Espinosa J. C. (2017). Examining the relationship between psychological capital and entrepreneurial intention: an exploratory study. Asian Soc. Sci. 13, 80–88. doi: 10.5539/ass.v13n3p80

[ref12] Coviello N. E. Jones M. V. (2004). Methodological issues in international entrepreneurship research. J. Bus. Ventur. 19, 485–508. doi: 10.1016/j.jbusvent.2003.06.001

[ref13] Crane F. G. (2014). Measuring and enhancing dispositional optimism and entrepreneurial intent in the entrepreneurial classroom: a Bahamian study. J. Acad. Bus. Educ. 15, 94–104.

[ref14] Cui J. (2021). The impact of entrepreneurship curriculum with teaching models on sustainable development of entrepreneurial mindset among higher education students in China: the moderating role of the entrepreneurial climate at the institution. Sustainability. 13:7950. doi: 10.3390/su13147950

[ref15] Cui J. Sun J. Bell R. (2021). The impact of entrepreneurship education on the entrepreneurial mindset of college students in China: the mediating role of inspiration and the role of educational attributes. Int. J. Manage. Educ. 19:100296. doi: 10.1016/j.ijme.2019.04.001

[ref16] Dello Russo S. Stoykova P. (2015). Psychological capital intervention (PCI): a replication and extension. Hum. Resour. Dev. Q. 26, 329–347. doi: 10.1002/hrdq.21212

[ref17] Dimov D. (2017). Towards a qualitative understanding of human capital in entrepreneurship research. Int. J. Entrep. Behav. Res. 23, 210–227. doi: 10.1108/IJEBR-01-2016-0016

[ref18] Donaldson S. (2013). Psychological capital, work engagement and organisational commitment amongst call Centre employees in South Africa. J. Posit. Psychol. 5, 177–191. doi: 10.1080/17439761003790930

[ref19] Fayolle A. Gailly B. (2015). The impact of entrepreneurship education on entrepreneurial attitudes and intention: hysteresis and persistence. J. Small Bus. Manag. 53, 75–93. doi: 10.1111/jsbm.12065

[ref20] Fayolle A. Liñán F. (2014). The future of research on entrepreneurial intentions. J. Bus. Res. 67, 663–666.

[ref21] Franke N. Lüthje C. (2004). Entrepreneurial intentions of business students: a benchmarking study. Int. J. Innov. Technol. Manag. 01, 269–288. doi: 10.1142/S0219877004000209

[ref22] Gao Q. Xu J. Tao Z. Liu L. Wu C. (2020). Exploration and analysis on the psychological capital of entrepreneurship and the deviant innovation behavior of employees. Front. Psychol. 11:1880. doi: 10.3389/fpsyg.2020.01880 PMC7438952 32903895

[ref23] Gibb A. (2002). In pursuit of a new ‘enterprise’ and ‘entrepreneurship’ paradigm for learning: creative destruction, new values, new ways of doing things and new combinations of knowledge. Int. J. Manage. Rev. 4, 233–269. doi: 10.1111/1468-2370.00086

[ref24] Gieure C. Benavides-Espinosa M. M. Roig-Dobón S. (2020). The entrepreneurial process: the link between intentions and behavior. J. Bus. Res. 112, 541–548. doi: 10.1016/j.jbusres.2019.11.088

[ref25] Hair J. Ringle C. Sarstedt M. (2011). PLS-SEM: indeed a silver bullet. J. Mark. Theory Pract. 19, 139–152. doi: 10.2753/MTP1069-6679190202

[ref26] Hmieleski K. M. Carr J. C. (2008). The relationship between entrepreneur psychological capital and new venture performance. Front. Entrepreneurship. Res. 10:1071. doi: 10.3389/fpsyg.2019.01071

[ref27] Ho M. H. R. Uy M. A. Kang B. N. Chan K. Y. (2018). Impact of entrepreneurship training on entrepreneurial efficacy and alertness among adolescent youth. Front. Educ. 3:13. doi: 10.3389/feduc.2018.00013

[ref28] Jarvis P. (2006). Towards a Comprehensive Theory of Human Learning. New York: Routledge.

[ref10] Jin C. (2017). The effect of psychological capital on start-up intention among young start-up entrepreneurs: a cross-cultural comparison. Chin. Manag. Stud. 11, 707–729. doi: 10.1108/CMS-06-2017-0162

[ref29] Kautonen T. Gelderen M. V. Fink M. (2013). Robustness of the theory of planned behavior in predicting entrepreneurial intentions and actions. Entrep. Theory Pract. 39, 655–674. doi: 10.1111/etap.12056

[ref501] Kirkley W. W. (2016). Entrepreneurial behaviour: The role of values. Int. J. Entrep. Behav. Res. 22, 290–328. doi: 10.1108/IJEBR-02-2015-0042

[ref30] Kyrö P. (2015). To grow or not to grow? Entrepreneurship and sustainable development. Handbook. Entrepreneurship Sustain. Dev. Res. 8, 15–28.

[ref31] Lackéus M. (2014). An emotion-based approach to assessing entrepreneurial education. Int. J. Manage. Educ. 12, 374–396. doi: 10.1016/j.ijme.2014.06.005

[ref32] Liu F. Ma J. Li R. (2019). Which role model is more effective in entrepreneurship education? An investigation of storytelling on individual’s entrepreneurial intention. Front. Psychol. 10:837. doi: 10.3389/fpsyg.2019.00837 31068853 PMC6491568

[ref33] Luthans F. (2002). Positive organizational behavior: developing and managing psychological strengths. Acad. Manag. Exec. 16, 57–72. doi: 10.5465/ame.2002.6640181

[ref34] Luthans F. Avey J. B. Avolio B. J. Peterson S. (2010). The development and resulting performance impact of positive psychological capital. Hum. Resour. Dev. Q. 21, 41–67. doi: 10.1002/hrdq.20034

[ref35] Luthans F. Avey J. B. Patera J. L. (2008). Experimental analysis of a web-based training intervention to develop positive psychological capital. Acad. Manag. Learn. Educ. 7, 209–221. doi: 10.5465/amle.2008.32712618

[ref36] Luthans F. Avolio B. J. Avey J. B. Norman S. M. (2007). Positive psychological capital: measurement and relationship with performance and satisfaction. Pers. Psychol. 60, 541–572. doi: 10.1111/j.1744-6570.2007.00083.x

[ref37] Luthans F. Youssef-Morgan C. M. (2017). The annual review of organizational psychology and organizational behavior. Annu. Rev. Organ. Psychol. Organ. Behav. 4, 339–366. doi: 10.1146/annurev-orgpsych-032516-113324

[ref38] Ma H. Barbe F. T. Zhang Y. C. (2018). Can social capital and psychological capital improve the entrepreneurial performance of the new generation of migrant workers in China? Sustainability. 10:3964. doi: 10.3390/su10113964

[ref39] MacKenzie S. Podsakoff P. Podsakoff N. (2011). Construct measurement and validation procedures in MIS and behavioral research: integrating new and existing techniques. MIS Q. 35, 293–334. doi: 10.2307/23044045

[ref40] Mahfud T. Triyono M. B. Sudira P. Mulyani Y. (2020). The influence of social capital and entrepreneurial attitude orientation on entrepreneurial intentions: the mediating role of psychological capital. Eur. Res. Manag. Bus. Econ. 26, 33–39. doi: 10.1016/j.iedeen.2019.12.005

[ref41] Martin M. B. Kikooma J. F. Kibanja G. M. (2016). Psychological capital and the startup capital–entrepreneurial success relationship. J. Small Bus. Entrepreneurship. 28, 27–54. doi: 10.1080/08276331.2015.1132512

[ref42] Martin B. C. McNally J. J. Kay M. J. (2013). Examining the formation of human capital in entrepreneurship: a meta-analysis of entrepreneurship education outcomes. J. Bus. Ventur. 28, 211–224. doi: 10.1016/j.jbusvent.2012.03.002

[ref43] Marvel M. R. Davis J. L. Sproul C. R. (2016). Human capital and entrepreneurship research: a critical review and future directions. Entrep. Theory Pract. 40, 599–626. doi: 10.1111/etap.12136

[ref44] Maslakcı A. Sesen H. Surucu L. (2021). Multiculturalism, positive psychological capital and students’ entrepreneurial intentions. Educ. Training 63, 597–612. doi: 10.1108/ET-04-2020-0073

[ref45] Masten A. S. Cutuli J. J. Herbers J. E. Reed M. (2009). “Resilience in development,” in Oxford Handbook of Positive Psychology. 2nd *Edn*. eds. Lopez Snyder (New York: Oxford Univ. Press), 117–131.

[ref46] Nabi G. Lińán F. Fayolle A. Krueger N. Walmsley A. (2017). The impact of entrepreneurship education in higher education: a systematic review and research agenda. Acad. Manag. Learn. Edu. 16, 277–299. doi: 10.5465/amle.2015.0026

[ref47] Newman A. Ucbasaran D. Zhu F. Hirst G. (2014). Psychological capital: a review and synthesis. J. Organ. Behav. 35, S120–S138. doi: 10.1002/job.1916

[ref48] Nowiński W. Haddoud M. Lančarič D. Egerová D. Czeglédi C. (2019). The impact of entrepreneurship education, entrepreneurial self-efficacy and gender on entrepreneurial intentions of university students in the Visegrad countries. Stud. Higher Educ. 44, 361–379. doi: 10.1080/03075079.2017.1365359

[ref49] Nowiński W. Haddoud M. Y. Wach K. Schaefer R. (2020). Perceived public support and entrepreneurship attitudes: a little reciprocity can go a long way! J. Vocational Behav. 112, 541–548. doi: 10.1016/j.jbusres.2019.11.088

[ref50] Ployhart R. E. Moliterno T. P. (2011). Emergence of the human capital resource: a multilevel model. Acad. Manag. Rev. 36, 127–150. doi: 10.5465/amr.2009.0318

[ref51] Podsakoff P. M. MacKenzie S. B. Lee J.-Y. Podsakoff N. P. (2003). Common method biases in behavioral research: a critical review of the literature and recommended remedies. J. Appl. Psychol. 88, 879–903. doi: 10.1037/0021-9010.88.5.879 14516251

[ref52] Rauch A. Hulsink W. (2015). Putting entrepreneurship education where the intention to act lies: an investigation into the impact of entrepreneurship education on entrepreneurial behavior. Acad. Manag. Learn. Edu. 14, 187–204. doi: 10.5465/amle.2012.0293

[ref53] Schmiedel T. Brocke J. V. Recker J. (2014). Development and validation of an instrument to measure organizational cultures’ support of business process management. Inf. Manag. 51, 43–56. doi: 10.1016/j.im.2013.08.005

[ref54] Seligman M. (1998). Learned Optimism. New York: Pocket Books.

[ref55] Shirokova G. Osiyevskyy O. Bogatyreva K. (2016). Exploring the intention-behavior link in student entrepreneurship: moderating effects of individual and environmental characteristics. Eur. Manag. J. 34, 386–399. doi: 10.1016/j.emj.2015.12.007

[ref56] Sieger P. Fueglistaller U. Zellweger T. (2014). Student Entrepreneurship Across the Globe: A Look at Intentions and Activities. St. Gallen: Swiss Research Institute of Small Business and Entrepreneurship at the University of St. Gallen (KMU-HSG).

[ref57] Sinclair V. G. Wallston K. A. (2004). The development and psychometric evaluation of the brief resilient coping scale. Assessment 11, 94–101. doi: 10.1177/1073191103258144, PMID: 14994958

[ref58] Snyder C. R. Irving L. Anderson J. (1991). “Hope and health: measuring the will and the ways,” in Handbook of Social and Clinical Psychology. eds. Snyder C. R. Forsyth D. R. (Elmsford, NY: Pergamon), 285–305.

[ref59] Souitaris V. Zerbinati S. Al-Laham A. (2007). Do entrepreneurship programmes raise entrepreneurial intention of science and engineering students? The effect of learning, inspiration and resources. J. Bus. Ventur. 22, 566–591. doi: 10.1016/j.jbusvent.2006.05.002

[ref60] Stajkovic A. D. Luthans F. (1998). Self-efficacy and work-related performance a meta-analysis. Psychol. Bull. 124, 240–261. doi: 10.1037/0033-2909.124.2.240

[ref62] Tang J.-J. (2020). Psychological capital and entrepreneurship sustainability. Front. Psychol. 11:866. doi: 10.3389/fpsyg.2020.00866 PMC7248200 32528347

[ref63] Tsai F.-S. Leonard K. M. Srivastava S. (2020). Editorial: the role of psychological capital in entrepreneurial contexts. Front. Psychol. 11:582133. doi: 10.3389/fpsyg.2020.582133 PMC7793970 33424695

[ref601] Unger J. M. Rauch A. Frese M. Rosenbusch N. (2011). Human capital and entrepreneurial success: A meta-analytical review. J. Bus. Ventur. 26, 341–358. doi: 10.1016/j.jbusvent.2012.03.002

[ref64] Wang Y. Tsai C.-H. Lin D. D. Enkhbuyant O. Cai J. (2019). Effects of human, relational, and psychological capitals on new venture performance. Front. Psychol. 10:1071. doi: 10.3389/fpsyg.2019.01071 PMC6591455 31275186

[ref65] Wei J. Chen Y. Zhang J. Gong Y. (2019). Research on factors affecting the entrepreneurial learning from failure: an interpretive structure model. Front. Psychol. 10:1304. doi: 10.3389/fpsyg.2019.01304 31214094 PMC6558073

[ref66] Xu W. Zhao S. (2020). The influence of entrepreneurs’ psychological capital on their deviant innovation behavior. Front. Psychol. 11:1606. doi: 10.3389/fpsyg.2020.01606 PMC7485554 32982813

[ref67] Youssef C. M. Luthans F. (2013). “Developing psychological capital in organizations: cognitive, affective and conative contributions of happiness,” in Oxford Handbook of Happiness. eds. David S. A. Boniwell I. Ayers A. C. (New York: Oxford Univ. Press), 751–766.

[ref68] Youssef-Morgan C. M. Luthans F. (2013). “Psychological capital theory: toward a positive holistic model,” in Advances in Positive Organizational Psychology, Vol. 19. ed. Bakker A. B. (Bingley, UK: Emerald), 145–166.

[ref69] Yueh H.-P. Wu Y. J. Chen W.-F. (2020). Editorial: the psychology and education of entrepreneurial development. Front. Psychol. 11:27. doi: 10.3389/fpsyg.2020.00027 32038440 PMC6992604

[ref70] Zeffane R. (2013). Need for achievement, personality and entrepreneurial potential: a study of young adults in the United Arab Emirates. J. Enterprising Cult. 21, 75–105. doi: 10.1142/S0218495813500040

[ref71] Zhao J. Wei G. Chen K.-H. Yien J.-M. (2020). Psychological capital and university students’ entrepreneurial intention in China: mediation effect of entrepreneurial capitals. Front. Psychol. 10:2984. doi: 10.3389/fpsyg.2019.02984 32038375 PMC6989491

